# Local fat, local risk: mesorectal fat volume outperforms BMI in predicting rectal cancer surgery outcomes

**DOI:** 10.3389/fnut.2026.1878649

**Published:** 2026-09-16

**Authors:** Tingting Liu, Liqiang Chang, Junrui Zhao, Yongjun Yu, Chao Chen, Mingsen Li, Zhao Zhang, Peng Huang, Shiwu Zhang, Hong Wang

**Affiliations:** 1 Department of Colorectal Surgery, Tianjin Union Medical Center, The First Affiliated Hospital of Nankai University, Tianjin, China; 2 Department of Anorectal Surgery, Shanxi Provincial Hospital of Traditional Chinese Medicine, Shanxi, China; 3 Department of Proctology, Binhai New Area Hospital of Traditional Chinese Medicine, Tianjin, China; 4 Nankai University School of Medicine, Nankai University, Tianjin, China; 5 Department of Pathology, Tianjin Union Medical Center, The First Affiliated Hospital of Nankai University, Tianjin, China; 6 First Teaching Hospital of Tianjin University of Traditional Chinese Medicine, Tianjin, China; 7 National Clinical Research Center for Chinese Medicine, Tianjin, China

**Keywords:** mesorectum, post-operative complications, rectal neoplasms, risk assessment, tomography

## Abstract

**Background:**

Accurate pre-operative assessment of surgical difficulty and post-operative risk in rectal cancer remains challenging. Conventional indices such as body mass index (BMI) fail to adequately reflect the pelvic surgical anatomy due to their systemic nature. Mesorectal fat volume—an imaging-derived, site-specific parameter—may better represent the operative field and associated risks, while also serving as a marker of regional adiposity relevant to nutritional and metabolic status.

**Methods:**

This single-center retrospective cohort study (2018–2026) analyzed 450 consecutive patients undergoing elective rectal cancer surgery with total mesorectal excision. Mesorectal fat volume was quantified using pre-operative computed tomography or magnetic resonance imaging with semi-automated segmentation. Patients were categorized into quartiles based on mesorectal fat volume. Operative outcomes and 30-day post-operative complications were compared across quartiles. Multivariable linear and logistic regression analyses were performed to identify independent predictors. Receiver operating characteristic (ROC) curve analysis was used to evaluate the discriminative performance of mesorectal fat volume, BMI, and pelvic index.

**Results:**

A total of 450 patients were stratified into quartiles according to mesorectal fat volume (MFV). Patients in the highest MFV quartile had significantly longer operative times (182.26 ± 9.28 vs. 163.03 ± 9.67 min, *P* < 0.001), greater intraoperative blood loss (169.63 ± 39.00 vs. 94.55 ± 40.08 mL, *P* < 0.001), higher conversion-to-open surgery rates (27.68% vs. 5.17%, *P* < 0.001), and higher 30-day post-operative complication rates (63.39% vs. 29.31%, *P* < 0.001) than those in the lowest MFV quartile. In multivariable analyses, MFV remained an independent predictor of post-operative complications (OR = 1.37, 95% CI: 1.21–1.55, *P* < 0.001), whereas BMI and pelvic index were not independently associated with complications. The multivariable prediction model demonstrated moderate discriminative ability (AUC = 0.683). Among the individual anthropometric measures, MFV demonstrated moderate discriminative ability (AUC = 0.655; 95% CI, 0.605–0.705), outperforming BMI (AUC = 0.552; 95% CI, 0.499–0.606) and pelvic index (AUC = 0.468; 95% CI, 0.415–0.522).

**Conclusion:**

CT-derived mesorectal fat volume is independently associated with increased operative difficulty and a higher risk of post-operative complications following total mesorectal excision. Compared with BMI and pelvic index, MFV provides better, although moderate, discrimination for predicting post-operative complications and may serve as a useful imaging biomarker for pre-operative risk stratification. Incorporation of MFV into multivariable prediction models may improve individualized surgical planning and perioperative risk assessment.

## Introduction

Rectal cancer surgery, particularly total mesorectal excision (TME), remains one of the most technically demanding procedures in colorectal surgery (1, 2). Despite advances in minimally invasive approaches and perioperative care, substantial variability persists in operative difficulty and post-operative outcomes (3, 4). This variability is not fully explained by tumor-related characteristics alone, highlighting the importance of patient-specific anatomical factors in determining surgical complexity and risk (5).

Obesity has long been considered a potential contributor to adverse surgical outcomes, with body mass index (BMI) commonly used as a surrogate marker (6, 7). However, BMI is a global and indirect measure of adiposity that fails to account for fat distribution or the specific anatomical constraints within the pelvis (8). Accumulating evidence suggests that BMI is an inconsistent predictor of operative difficulty and post-operative complications in rectal cancer surgery (9). Similarly, pelvic anatomical indices have been proposed to estimate technical difficulty, but their predictive accuracy has been limited and inconsistent (10). These limitations underscore the need for a more precise, localized measure of adiposity within the surgical field.

In recent years, there has been growing interest in imaging-derived biomarkers that more precisely reflect the local surgical environment (11–13). Mesorectal fat volume, quantified using pre-operative computed tomography (CT) or magnetic resonance imaging (MRI), represents a direct and localized measure of adiposity within the mesorectal compartment (14, 15). These factors can translate into prolonged operative time, increased blood loss, higher conversion rates, and an elevated risk of post-operative complications, including anastomotic leakage and surgical site infection (16–20). Neoadjuvant treatment may alter pelvic tissue characteristics through fibrosis and inflammatory changes; however, its specific interaction with mesorectal fat volume was not evaluated in the present study and warrants future investigation.

Despite growing interest, the existing literature on mesorectal fat and surgical outcomes remains limited (21, 22). Prior studies are characterized by small sample sizes, single center designs, and heterogeneous methodologies, restricting generalizability (23). Few studies have simultaneously evaluated mesorectal fat volume alongside conventional metrics within a comprehensive analytical framework (24), and comparative assessments using multivariable modeling and ROC analysis are scarce (25). Consequently, the incremental clinical value of mesorectal fat volume over traditional indices remains insufficiently defined (22, 26).

Addressing these gaps is essential for improving pre-operative risk stratification and surgical planning. A more accurate anatomical biomarker could facilitate individualized surgical approaches, optimize resource allocation, and ultimately improve patient outcomes.

Therefore, the present study aimed to investigate the association between mesorectal fat volume and operative as well as post-operative outcomes in a large cohort of patients undergoing rectal cancer surgery. In addition, we sought to compare its predictive performance with BMI and pelvic index using multivariable regression and ROC curve analysis. We hypothesized that mesorectal fat volume would serve as a superior predictor of surgical complexity and 30-day post-operative complications compared with conventional anthropometric and anatomical measures.

## Methods

### Study design

This retrospective observational cohort study was conducted in accordance with the STROBE Statement. Patients were included between January 2018 and February 2026. The aim was to evaluate the association between mesorectal fat volume and perioperative outcomes in patients undergoing rectal cancer surgery, and to compare its predictive performance with conventional anthropometric and anatomical indices. This study was conducted at a tertiary referral colorectal surgery center.

### Ethical considerations

The study protocol was approved by the Institutional Ethics Committee of Tianjin People's Hospital [Approval No. (2025) Lun-Shen (C24)], and all procedures were conducted in accordance with the principles of the Declaration of Helsinki. Due to the retrospective design and use of anonymized data, the requirement for informed consent was waived.

### Study population

A total of 450 consecutive patients who underwent elective rectal cancer resection with curative intent were included. Patients were identified from a prospectively maintained institutional database. Eligible participants had histologically confirmed rectal adenocarcinoma and available pre-operative cross-sectional imaging suitable for quantitative analysis.

Patients undergoing emergency procedures, those with recurrent disease, or those missing key exposure or outcome variables (>10% missingness threshold) were excluded. All procedures were performed by seven fellowship-trained colorectal surgeons at a tertiary referral center. Each surgeon had more than 5 years of independent colorectal surgical practice and maintained an annual caseload exceeding 40 TME procedures. Surgical technique followed standardized total mesorectal excision principles and institutional treatment protocols.

All surgical procedures were performed at a tertiary referral colorectal surgery center according to standardized TME principles and institutional treatment protocols. Surgical approaches included laparoscopic, robotic, and open procedures. Of the 450 included patients, 320 (71.1%) underwent laparoscopic TME, 84 (18.7%) underwent robotic TME, and 46 (10.2%) underwent open TME. Surgical procedures included low anterior resection (*n* = 309, 68.7%), intersphincteric resection (*n* = 45, 10.0%), and abdominoperineal resection (*n* = 96, 21.3%). Tumor height was categorized according to distance from the anal verge as low (< 5 cm; *n* = 177, 39.3%), mid (5–10 cm; *n* = 206, 45.8%), and upper (>10 cm; *n* = 67, 14.9%).

Although this was a retrospective study including all eligible patients during the study period, a *post-hoc* sample size adequacy assessment was performed. Assuming a baseline complication rate of approximately 30% and an expected odds ratio of ≥1.8 for increased mesorectal fat volume, a minimum sample of approximately 380 patients would provide 80% power at a two-sided alpha level of 0.05. Therefore, the final sample size of 450 patients was considered sufficient to detect clinically meaningful differences and to support multivariable modeling without overfitting.

### Data collection

Demographic, clinical, and perioperative variables were extracted from electronic medical records. These included age, sex, body mass index (BMI), tumor stage, receipt of neoadjuvant therapy, operative time, intraoperative blood loss, conversion to open surgery, and surgeon-reported operative difficulty score. Post-operative outcomes were assessed within 30 days and included overall complications, anastomotic leakage, and surgical site infection, defined according to standard clinical criteria.

### Imaging acquisition and mesorectal fat segmentation

Pre-operative imaging was performed using contrast-enhanced computed tomography (CT) or high-resolution pelvic magnetic resonance imaging (MRI) according to institutional protocols. For CT, scans were acquired with patients in the supine position using multidetector scanners with a slice thickness of 3–5 mm. MRI examinations included T2-weighted sequences in axial, sagittal, and coronal planes with high spatial resolution optimized for pelvic evaluation (27).

In this cohort, pre-operative imaging comprised contrast enhanced CT in 312 patients (69.3%) and high resolution pelvic MRI in 138 patients (30.7%). To assess comparability between modalities, mesorectal fat volume measurements were evaluated separately by imaging type. Mean mesorectal fat volume did not differ significantly between CT and MRI groups (CT: 312 ± 42 cm^3^ vs. MRI: 320 ± 38 cm^3^; *p* = 0.21). Furthermore, the predictive performance of mesorectal fat volume for 30 day post-operative complications was consistent across modalities [CT subgroup AUC = 0.71 [95% CI: 0.66–0.75] vs. MRI subgroup AUC = 0.73 [95% CI: 0.67–0.77]; *p* for interaction = 0.38]. These findings support the comparability of mesorectal fat volume measurements derived from CT and MRI using the standardized segmentation protocol described below.

Mesorectal fat volume was quantified using a semi-automated segmentation approach implemented in 3D Slicer software (version 5.6.2, Brigham and Women's Hospital, Boston, MA, USA), an open-source medical image analysis platform widely used for volumetric segmentation. The mesorectal compartment was defined anatomically as the adipose tissue enclosed within the mesorectal fascia, extending from the level of the sacral promontory superiorly to the levator ani muscles inferiorly.

Segmentation was performed on axial slices. The mesorectal fascia was manually delineated as the outer boundary, while the rectal wall constituted the inner boundary. Within this region of interest, adipose tissue was identified based on standardized radiological criteria. For CT images, fat voxels were selected using attenuation thresholds typically ranging from −190 to −30 Hounsfield units. For MRI, fat-containing regions were identified based on high signal intensity on T2-weighted images, with additional confirmation using fat-suppressed sequences when available. Following segmentation, the total mesorectal fat volume was computed by summing voxel volumes across all slices, yielding a three-dimensional volumetric measurement expressed in cubic centimeters (cm^3^). All segmentations were performed by trained investigators blinded to clinical outcomes, and a subset of cases was independently reviewed to ensure consistency. Interobserver variability was minimized through standardized protocols and calibration sessions prior to analysis. To further enhance measurement reliability, interobserver and intraobserver agreement were evaluated in a random subset of 50 patients using intraclass correlation coefficients (ICCs). An ICC >0.85 was considered indicative of excellent agreement. The intraclass correlation coefficient was 0.91 (95% CI: 0.86–0.95) for interobserver agreement and 0.93 (95% CI: 0.89–0.96) for intraobserver agreement. A two-way random-effects model (absolute agreement) was used. For statistical analysis, mesorectal fat volume was examined both as a continuous variable, scaled per 50 cm^3^ increment, and as a categorical variable divided into quartiles (Q1–Q4) based on its distribution within the study population (28–30).

### Pelvic index measurement

Pelvic dimensions were measured on pre-operative imaging using predefined anatomical landmarks. The pelvic index was calculated as a ratio reflecting the geometric configuration of the bony pelvis relevant to surgical access. Measurements were obtained on standardized axial and sagittal planes to ensure reproducibility. All pelvic measurements were performed independently by two observers, and mean values were used for analysis to reduce measurement error. The pelvic index was calculated as the ratio of pelvic inlet anteroposterior diameter to intertuberous distance on standardized axial and sagittal planes.

### Outcomes

The primary outcomes were operative parameters reflecting surgical difficulty, including operative time, intraoperative blood loss, conversion to open surgery, and difficulty score. Operative difficulty was assessed immediately after surgery using a standardized 10 point scale completed by the operating surgeon. This scoring system was adapted from a previously validated instrument described by Yuval et al. (31), with established content validity based on expert consensus on determinants of operative complexity in rectal cancer surgery. Scores of 1–3 represented minimal technical difficulty with clear visualization and uncomplicated dissection; scores of 4–6 represented moderate difficulty requiring additional technical maneuvers or more demanding pelvic dissection; and scores of 7–10 represented severe technical difficulty characterized by restricted visualization, limited instrument maneuverability, challenging pelvic anatomy, or repeated disruption of tissue planes (31). Prior to study initiation, participating surgeons underwent departmental calibration sessions using representative operative videos to promote consistency in scoring practices. To assess interrater reliability, a random subset of 30 operative videos was independently scored by two additional colorectal surgeons blinded to the primary surgeon's assessment. The intraclass correlation coefficient (ICC) for the operative difficulty score was 0.84 (95% CI: 0.72–0.92), indicating good to excellent interrater agreement. Construct validity was further supported by significant positive correlations between difficulty scores and objective intraoperative measures, as presented in the Results section (operative time: Spearman *r* = 0.472, *p* < 0.001; blood loss: Spearman *r* = 0.466, *p* < 0.001). The main post-operative endpoint was the occurrence of any complication within 30 days. Secondary outcomes included anastomotic leakage and surgical site infection. Post-operative complications were additionally classified according to the Clavien–Dindo grading system, and sensitivity analyses were performed considering major complications (grade ≥III) as a separate endpoint (32).

### Statistical analysis

Continuous variables are presented as mean ± standard deviation, while categorical variables are expressed as frequencies and percentages. Prior to the final statistical analysis, the dataset underwent a structured data-quality review. Potentially anomalous observations were initially flagged using distributional and interquartile-range screening and were subsequently reviewed at the individual-record level against the corresponding source information. Statistical extremeness or deviation from normality alone was not considered sufficient justification for modification of an observation. Four records identified during this verification process were determined to contain data-quality anomalies and were corrected before the final analysis. No participant was excluded solely because of an extreme value. Comparisons across quartiles of mesorectal fat volume were performed using one-way analysis of variance (ANOVA) for continuous variables and the chi-square test for categorical variables. A two-sided *p*-value < 0.05 was considered statistically significant. Multivariable linear regression analysis was conducted to assess independent predictors of operative time. Multivariable logistic regression analysis was used to evaluate factors associated with 30-day post-operative complications. Results are reported as β coefficients or odds ratios (ORs) with corresponding 95% confidence intervals. Multicollinearity among covariates was assessed using variance inflation factors (VIF), with a threshold of < 5 considered acceptable. Model calibration was evaluated using the Hosmer–Lemeshow goodness-of-fit test, and discrimination was assessed using the c-statistic. The discriminative performance of mesorectal fat volume, BMI, and pelvic index for predicting post-operative complications was assessed using receiver operating characteristic (ROC) curve analysis. The area under the curve (AUC) was calculated for each predictor, with higher values indicating better discrimination. In addition, model calibration was evaluated using calibration plots comparing predicted and observed probabilities of post-operative complications. Decision Curve Analysis (DCA) was performed to assess the clinical utility and net benefit of incorporating mesorectal fat volume into risk prediction across a range of clinically relevant threshold probabilities. These analyses were conducted in accordance with contemporary recommendations for evaluation of clinical prediction models. A sensitivity analysis excluding the first 20 TME cases performed by each surgeon during the study period was conducted to evaluate the potential influence of learning-curve effects. To account for potential surgical heterogeneity, multivariable regression models were additionally adjusted for surgical approach.

## Results

### Baseline characteristics

A total of 450 patients who underwent total mesorectal excision (TME) for rectal cancer were included in the study. The overall surgical characteristics of the study cohort, including surgical approach, procedure type, tumor height category, neoadjuvant treatment, and annual TME caseload, are summarized in Supplementary Table S1. Patients were subsequently categorized into four quartiles according to mesorectal fat volume (MFV): Q1 (*n* = 116), Q2 (*n* = 109), Q3 (*n* = 113), and Q4 (*n* = 112). Baseline demographic and clinicopathological characteristics according to MFV quartiles are presented in Table 1.

**Table 1:** Baseline characteristics according to mesorectal fat volume quartiles.

Variable	Q1 (*n* = 116)	Q2 (*n* = 109)	Q3 (*n* = 113)	Q4 (*n* = 112)	*P-*value
Age, years	62.22 ± 10.61	62.72 ± 9.48	62.19 ± 9.98	62.89 ± 10.53	0.938
Male sex, *n* (%)	69 (59.48)	66 (60.55)	74 (65.49)	65 (58.04)	0.685
BMI, kg/m^2^	26.51 ± 3.58	26.82 ± 3.85	26.91 ± 3.47	27.63 ± 4.10	0.146
Tumor stage, *n* (%)					0.775
II	45 (38.79)	43 (39.45)	46 (40.71)	42 (37.50)	
III	51 (43.97)	53 (48.62)	56 (49.56)	54 (48.21)	
IV	20 (17.24)	13 (11.93)	11 (9.73)	16 (14.29)	
Neoadjuvant therapy, *n* (%)	53 (45.69)	54 (49.54)	58 (51.33)	56 (50.00)	0.847
Pelvic index	0.99 ± 0.13	1.00 ± 0.14	1.01 ± 0.15	0.99 ± 0.14	0.578
Mesorectal fat volume, cm^3^	217.53 ± 34.98	290.72 ± 16.18	351.00 ± 16.59	434.54 ± 45.32	< 0.001

BMI, body mass index; SD, standard deviation. Continuous variables are presented as mean ± SD and were compared using one-way ANOVA. Categorical variables are presented as number (%) and were compared using the Pearson chi-square test.

There were no significant differences among the four groups with respect to age, sex, BMI, tumor stage, neoadjuvant therapy, annual TME volume, surgical approach, procedure type, or tumor height category (all *P* > 0.05).

### Perioperative outcomes

Perioperative outcomes according to MFV quartiles are summarized in Table 2. Increasing MFV was associated with significantly longer operative time (163.03 ± 9.67, 170.61 ± 8.61, 174.11 ± 10.39, and 182.26 ± 9.28 min, respectively; *P* < 0.001). Mean estimated blood loss also increased progressively from 94.55 ± 40.08 mL in Q1 to 169.63 ± 39.00 mL in Q4 (*P* < 0.001).

**Table 2:** Operative outcomes according to mesorectal fat volume quartiles.

Outcome	Q1 (*n* = 116)	Q2 (*n* = 109)	Q3 (*n* = 113)	Q4 (*n* = 112)	*P-*value
Operative time, min	163.03 ± 9.67	170.61 ± 8.61	174.11 ± 10.39	182.26 ± 9.28	< 0.001
Blood loss, mL	94.55 ± 40.08	111.18 ± 34.57	133.98 ± 37.19	169.63 ± 39.00	< 0.001
Conversion to open surgery, *n* (%)	6 (5.17)	21 (19.27)	29 (25.66)	31 (27.68)	< 0.001
Surgical difficulty score	3.80 ± 0.90	4.77 ± 0.96	5.99 ± 1.00	6.99 ± 1.27	< 0.001

Continuous variables are presented as mean ± SD and compared using one-way ANOVA. Categorical variables are presented as number (%) and compared using the Pearson chi-square test.

Likewise, the conversion-to-open surgery rate increased significantly across quartiles (5.17%, 19.27%, 25.66%, and 27.68%, respectively; *P* < 0.001).

Surgical difficulty scores also increased significantly with increasing MFV (*P* < 0.001), indicating that greater mesorectal fat volume was associated with technically more demanding procedures.

### Post-operative outcomes

Post-operative outcomes are presented in Table 3. The overall 30-day post-operative complication rate increased significantly with increasing MFV, from 29.31% in Q1 to 63.39% in Q4 (*P* < 0.001). Overall, 213 of 450 patients (47.33%) experienced at least one complication.

**Table 3:** Thirty-day post-operative outcomes according to mesorectal fat volume quartiles.

Outcome	Q1 (*n* = 116)	Q2 (*n* = 109)	Q3 (*n* = 113)	Q4 (*n* = 112)	*P-*value
Any post-operative complication, *n* (%)	34 (29.31)	52 (47.71)	56 (49.56)	71 (63.39)	< 0.001
Anastomotic leakage, *n* (%)	8 (6.90)	7 (6.42)	16 (14.16)	15 (13.39)	0.131
Surgical site infection, *n* (%)	17 (14.66)	15 (13.76)	20 (17.70)	33 (29.46)	0.009
Reoperation, *n* (%)	5 (4.31)	12 (11.01)	18 (15.93)	21 (18.75)	0.006

Categorical variables are presented as number (%) and compared using the Pearson chi-square test. Post-operative complications were assessed within 30 days after surgery.

Anastomotic leak rates demonstrated an increasing numerical trend (6.90%, 6.42%, 14.16%, and 13.39%, respectively); however, this difference did not reach statistical significance (*P* = 0.131). Surgical site infection and reoperation rates increased significantly across MFV quartiles. Among the 56 patients who required reoperation, 44 (78.6%) were classified as Clavien–Dindo grade III and 12 (21.4%) as grade IV; no reoperations occurred in grades I or II. It is important to note that the total complication rate reflects the number of patients who experienced at least one complication, and the individual complication types (e.g., anastomotic leak, SSI) are not mutually exclusive, as patients may experience multiple events.

### Multivariable analyses

The results of the multivariable regression analyses are presented in Table 4. In the multivariable linear regression model, mesorectal fat volume remained independently associated with operative time (β = 4.24, *P* < 0.001), whereas BMI and pelvic index were not significant independent predictors.

**Table 4:** Multivariable regression analyses.

A. Multivariable linear regression for operative time			
Variable	β coefficient	95% CI	*P-*value
Mesorectal fat volume (per 50 cm^3^)	4.24	3.78–4.69	< 0.001
BMI (kg/m^2^)	−0.04	−0.25 to 0.17	0.703
Age (years)	0.04	−0.04 to 0.11	0.361
Male sex	5.95	4.35–7.56	< 0.001
Stage III–IV	6.27	4.68–7.86	< 0.001
Pelvic index	−0.02	−5.43 to 5.40	0.995
Model performance: *R*^2^ = 0.503; adjusted *R*^2^ = 0.497; *F*_(6, 443)_ = 74.82; *P* < 0.001.			
B. Multivariable logistic regression for 30-day post-operative complications			
Variable	Odds ratio (OR)	95% CI	*P-*value
Mesorectal fat volume (per 50 cm^3^)	1.37	1.21–1.55	< 0.001
BMI (kg/m^2^)	1.03	0.97–1.08	0.340
Age (years)	1.01	0.99–1.03	0.319
Male sex	1.35	0.90–2.02	0.153
Stage III–IV	1.58	1.06–2.37	0.026
Pelvic index	0.32	0.08–1.27	0.106
Conversion to open surgery	1.87	1.12–3.12	0.016
Model performance: Hosmer–Lemeshow *p* = 0.444, c-statistic (AUC) = 0.683, LR $\chi_{\left(7\right)}^{2}$ = 48.26, *p* < 0.001, Pseudo *R*^2^ = 0.078.			

In the multivariable logistic regression model, mesorectal fat volume remained independently associated with 30-day post-operative complications (OR = 1.37, 95% CI: 1.21–1.55, *P* < 0.001) after adjustment for age, sex, BMI, pelvic index, tumor stage, and conversion to open surgery. The model demonstrated moderate discriminative ability (AUC = 0.683) with limited explanatory power (Pseudo R^2^ = 0.078).

### Predictive performance

The predictive performance of the multivariable model and individual anthropometric parameters is summarized in Table 5.

**Table 5:** Receiver operating characteristic (ROC) analysis for predicting 30-day post-operative complications.

Predictor	AUC (95% CI)	Optimal cutoff	Sensitivity (%)	Specificity (%)	PPV (%)	NPV (%)
Mesorectal fat volume	**0.655 (0.605–0.705)**	290 cm^3^	76.28	47.66	57.14	68.71
BMI	**0.552 (0.499–0.606)**	29.1 kg/m^2^	29.52	75.00	53.78	57.92
Pelvic index	**0.468 (0.415–0.522)**	1.08	29.52	66.67	49.33	56.45

AUC, area under the curve; CI, confidence interval; PPV, positive predictive value; NPV, negative predictive value. Optimal cutoff values were determined using the maximum Youden index. Mesorectal fat volume demonstrated significantly greater discriminative ability than BMI (*P* = 0.004) and pelvic index (*P* < 0.001) according to DeLong's test. Bold values indicate statistically significant results.

The receiver operating characteristic (ROC) curve for the multivariable prediction model is presented in Figure 1, demonstrating moderate discriminative ability for predicting 30-day post-operative complications. The ROC curves comparing the individual anthropometric measures are shown in Figure 2. Mesorectal fat volume demonstrated moderate discriminative ability (AUC = 0.655; 95% CI, 0.605–0.705), outperforming BMI (AUC = 0.552; 95% CI, 0.499–0.606) and pelvic index (AUC = 0.468; 95% CI, 0.415–0.522). Using an optimal cutoff value of 290 cm^3^ (based on the Youden index), MFV achieved a sensitivity of 76.28%, a specificity of 47.66%, a positive predictive value of 57.14%, and a negative predictive value of 68.71%, indicating moderate predictive performance.

**Figure 1:**
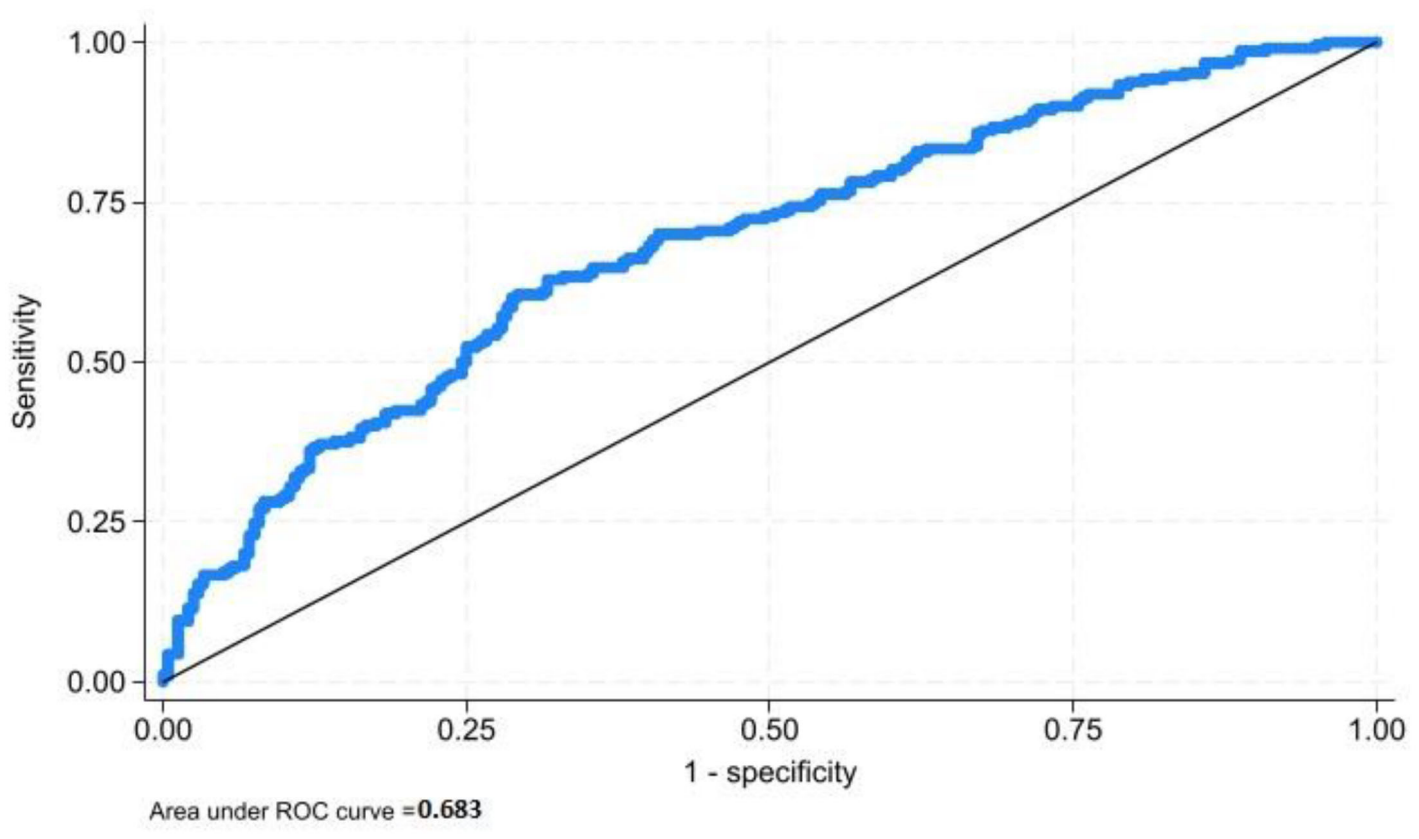
Receiver operating characteristic (ROC) curves for predicting 30-day post-operative complications.

**Figure 2:**
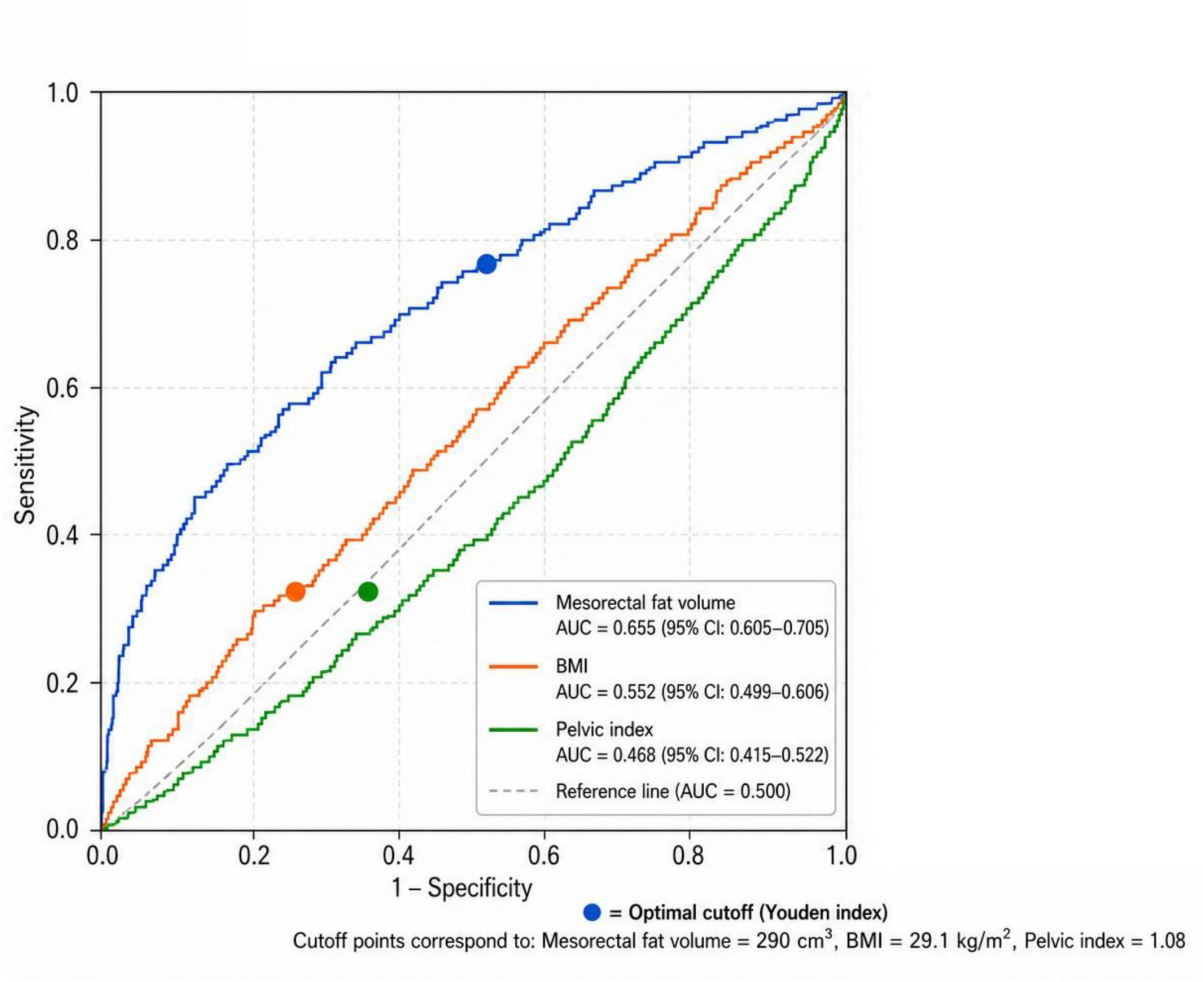
Receiver operating characteristic (ROC) curve for mesorectal fat volume predicting 30-day post-operative complications.

The calibration plot (Figure 3) demonstrated satisfactory agreement between predicted and observed probabilities of post-operative complications, indicating good calibration of the multivariable prediction model.

**Figure 3:**
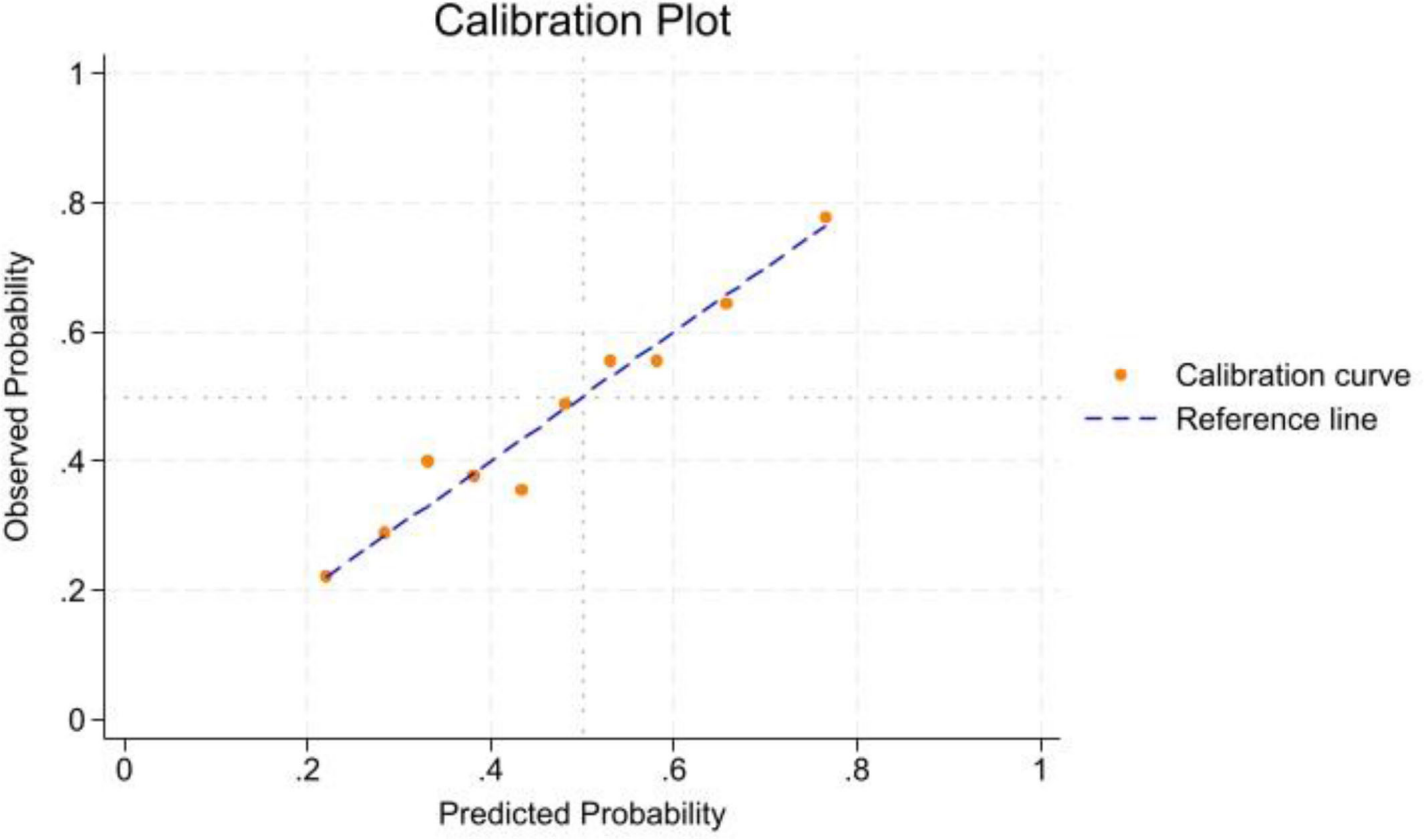
Calibration Plot for Prediction of 30-Day Post-operative Complications. Calibration plot comparing predicted and observed probabilities of 30-day post-operative complications. The diagonal reference line represents perfect agreement between predicted and observed risk. The calibration curve demonstrates satisfactory correspondence between model-predicted probabilities and actual complication rates across risk strata, indicating acceptable calibration of the multivariable prediction model.

The clinical utility of the multivariable model was further evaluated using decision curve analysis (Figure 4). The model demonstrated greater net clinical benefit than both the treat-all and treat-none strategies across a clinically relevant range of threshold probabilities, supporting the potential value of incorporating mesorectal fat volume into individualized pre-operative risk assessment.

**Figure 4:**
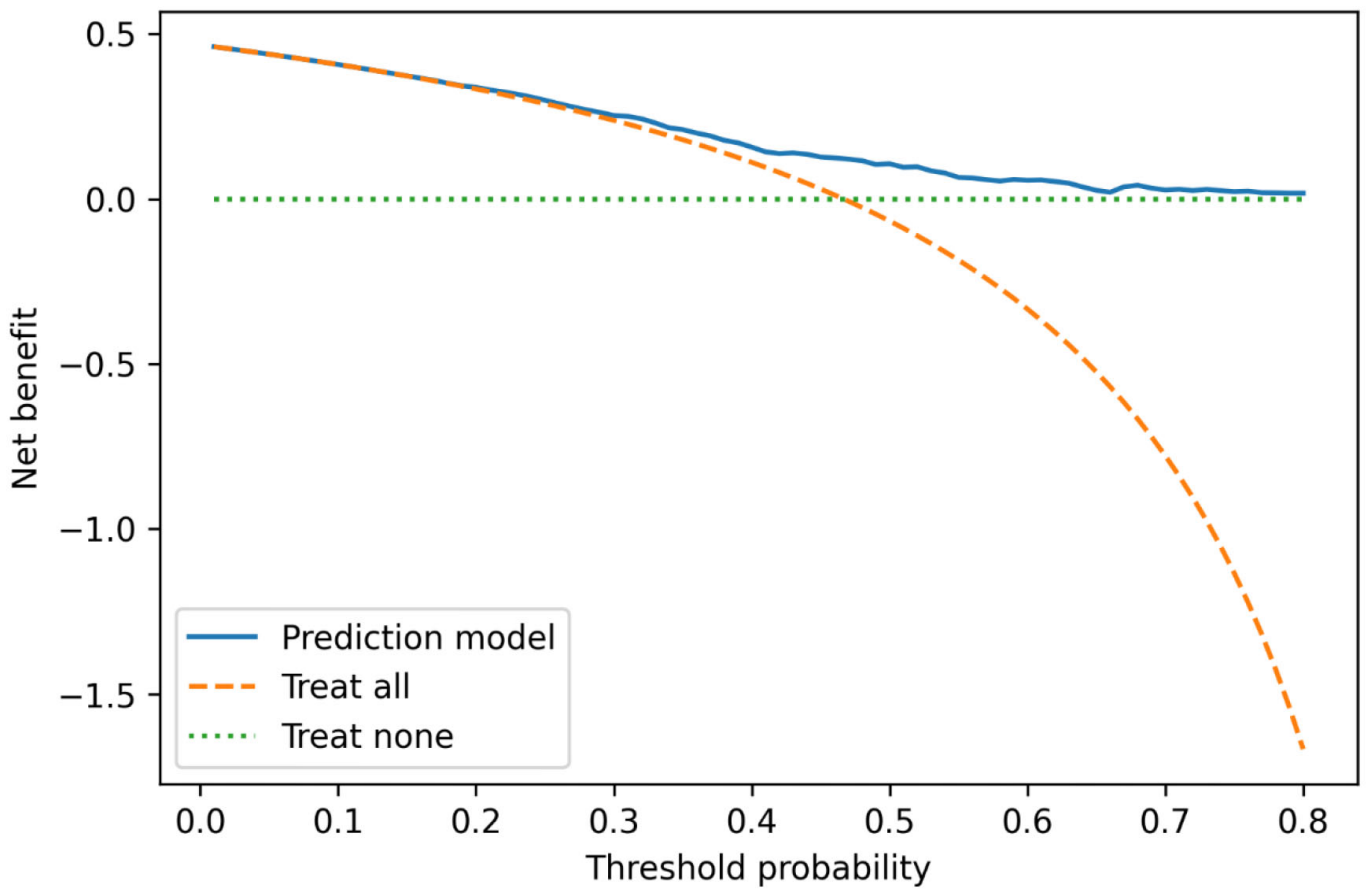
Decision curve analysis for prediction of 30-day post-operative complications.

## Discussion

In this study, we demonstrate that mesorectal fat volume is a significant, independent predictor of operative difficulty and short-term post-operative outcomes in rectal cancer surgery. Increasing mesorectal fat volume was consistently associated with prolonged operative time, increased blood loss, higher conversion rates, and elevated 30-day complication risk. Importantly, mesorectal fat volume demonstrated superior discriminatory performance compared with BMI and pelvic index (AUC = 0.655 vs. 0.552 and 0.468, respectively), although its overall discriminative ability was moderate. The multivariable prediction model further demonstrated moderate discrimination (AUC = 0.683), supporting the potential role of MFV as a clinically relevant imaging biomarker for pre-operative risk stratification. The progressive increase in overall post-operative complications across quartiles suggests a dose-response relationship. *This overall rate includes patients who experienced multiple complications, and the individual complication subtypes are not mutually exclusive*.

The association between mesorectal fat volume and operative complexity is biologically plausible (30, 32). Excess adipose tissue within the mesorectal compartment obscures surgical planes, reduces visibility, and limits instrument maneuverability within the confined pelvis (31, 33)—factors particularly relevant in minimally invasive approaches (15). The stepwise increase in operative time and difficulty scores across quartiles reflects this anatomical constraint, confirming that local fat distribution, rather than generalized obesity, is a key determinant of surgical challenge. This association remained significant after adjustment for surgical approach, tumor location, and procedure type, indicating that MFV captures anatomical characteristics not adequately reflected by conventional classifications. These findings are consistent with prior imaging-based studies (1, 6, 10, 30), though those were limited by small sample sizes and heterogeneous methods. Increased mesorectal adiposity may additionally impair traction-countertraction dynamics and tissue handling (34).

Our findings also extend prior observations by demonstrating a clear relationship between mesorectal fat volume and post-operative morbidity (20, 29). The progressive increase in overall post-operative complications and surgical site infection across quartiles suggests a dose-response relationship. Although an increasing trend in anastomotic leak rates was observed, this difference did not reach statistical significance (*P* = 0.131). This may be explained by multiple mechanisms, including technically demanding dissection leading to suboptimal tissue handling, impaired perfusion at the anastomotic site, and a pro-inflammatory milieu associated with increased adiposity. The independent association observed in multivariable analysis, even after adjustment for known confounders, reinforces the robustness of this relationship. In line with previous reports on visceral obesity and colorectal surgery, adipose tissue is metabolically active and may promote local inflammation, oxidative stress, and microvascular dysfunction, all of which can adversely affect wound healing and anastomotic integrity (35). However, unlike prior studies relying on indirect obesity measures, our volumetric approach provides a more precise quantification of the local adipose burden.

Although neoadjuvant treatment may influence pelvic tissue characteristics through fibrosis and inflammatory changes, potentially increasing operative complexity, mesorectal fat volume may provide complementary anatomical information beyond treatment status alone. Previous studies have reported that treatment-related fibrosis can impair visualization and dissection within the mesorectal plane, resulting in longer operative times and greater technical difficulty (36–38). However, neoadjuvant therapy was not independently associated with operative or post-operative outcomes in the present cohort. Future prospective multicenter studies should investigate potential interactions between treatment-related anatomical alterations and mesorectal fat volume.

A key contribution of this study is the comparative evaluation of mesorectal fat volume against BMI and pelvic index. While BMI has traditionally been used as a surrogate for obesity, it does not capture regional fat distribution or the anatomical constraints within the pelvis (9, 32, 33). In our analysis, BMI was not significantly associated with operative time or post-operative complications and demonstrated poor predictive performance in ROC analysis. Similarly, pelvic index showed limited discriminatory ability. In contrast, mesorectal fat volume consistently demonstrated superior predictive performance, with moderate discriminative ability (AUC = 0.655), outperforming BMI (AUC = 0.552) and pelvic index (AUC = 0.468), highlighting its potential utility for pre-operative risk stratification. These findings underscore the limitations of conventional metrics and support a shift toward imaging-based, region-specific biomarkers. Previous studies evaluating BMI and pelvic dimensions have reported inconsistent or weak associations with surgical outcomes, which may be attributable to their inability to reflect the true operative field (8); our findings corroborate and extend this concept by demonstrating the superiority of a direct volumetric parameter. While ROC analysis demonstrated that MFV provided superior discrimination compared with BMI and pelvic index, discrimination alone does not necessarily translate into improved clinical decision-making. Therefore, we additionally evaluated model calibration and clinical utility. Calibration analysis demonstrated satisfactory agreement between predicted and observed complication risks, supporting the reliability of risk estimates generated by the model. Furthermore, Decision Curve Analysis showed a favorable net benefit across clinically relevant threshold probabilities, suggesting that incorporation of MFV may improve pre-operative risk assessment and patient counseling. Although the model demonstrated satisfactory calibration and favorable clinical utility, its explanatory power was limited (Pseudo *R*^2^ = 0.078), indicating that post-operative complications are influenced by multiple patient-, tumor-, and procedure-related factors beyond mesorectal fat volume alone. These findings strengthen the potential clinical applicability of MFV as an imaging-derived biomarker for identifying patients at increased risk of technically challenging surgery and post-operative complications (35).

Surgeon-related factors are recognized determinants of operative complexity and post-operative outcomes in rectal cancer surgery (39, 40). In the present study, all procedures were performed by seven fellowship-trained colorectal surgeons using standardized total mesorectal excision principles, and surgeon characteristics (including years of experience and annual TME volume) were recorded. However, the limited number of surgeons precluded formal mixed-effects modeling with surgeon as a random effect. As an alternative, a sensitivity analysis excluding the first 20 TME cases performed by each surgeon during the study period was conducted; the associations between mesorectal fat volume and operative outcomes remained statistically significant, suggesting that learning-curve effects did not materially influence the observed results. While residual confounding by unmeasured surgeon-specific factors cannot be entirely excluded, the consistency of the findings across multiple objective endpoints and the robustness to exclusion of early cases support the independent predictive value of mesorectal fat volume. Future prospective studies with larger numbers of surgeons and more diverse caseloads should incorporate hierarchical models to further evaluate the independent contribution of mesorectal fat volume to surgical difficulty and post-operative outcomes, while accounting for surgeon-level clustering.

The results of this study address several important gaps in the existing literature. Previous investigations into mesorectal fat and surgical outcomes have been limited by small sample sizes, heterogeneous methodologies, and inconsistent findings. Few studies have incorporated comprehensive multivariable modeling alongside formal assessment of predictive performance. By leveraging a relatively large cohort and integrating volumetric imaging analysis with robust statistical methods, the present study provides more definitive evidence regarding the clinical relevance of mesorectal fat volume. Additionally, the incorporation of ROC curve analysis and direct comparison with established indices represents an important methodological advancement over prior work, allowing for a more objective evaluation of clinical utility.

Clinically, incorporation of MFV into pre-operative assessment may facilitate more accurate risk stratification and surgical planning. Patients with high MFV may benefit from assignment to more experienced surgical teams, consideration of alternative operative approaches, or enhanced perioperative optimization strategies. Integration of MFV into predictive models could further improve individualized risk estimation.

Several limitations should be acknowledged. First, the retrospective design introduces the potential for selection bias and residual confounding, despite multivariable adjustment. Second, although segmentation protocols were standardized, measurement of mesorectal fat volume may still be subject to observer variability and differences in imaging quality. Third, the study was conducted at a single center, which may limit generalizability to other populations or surgical settings. Fourth, only short-term outcomes were evaluated; the impact of mesorectal fat volume on long-term oncologic outcomes remains unclear. Fifth, although the prediction model demonstrated satisfactory calibration and clinical utility, its discriminative performance remained moderate (AUC = 0.683) and its explanatory power was limited (Pseudo R^2^ = 0.078). Therefore, mesorectal fat volume should be considered an adjunct to, rather than a replacement for, comprehensive pre-operative risk assessment. In addition, variations in imaging modality (CT vs. MRI) and segmentation techniques across institutions may influence reproducibility, highlighting the need for standardized protocols.

Future studies should aim to validate these findings in multicenter prospective cohorts and explore the integration of mesorectal fat volume into comprehensive predictive models, potentially incorporating advanced imaging techniques and machine learning approaches. Additionally, the relationship between mesorectal fat and long-term oncologic outcomes warrants further investigation. Further research is also needed to determine clinically relevant cutoff values and to assess whether modification of surgical strategy based on mesorectal fat volume can improve patient outcomes. Future studies should also evaluate whether incorporation of additional clinical, radiological, and operative variables can further improve predictive performance beyond mesorectal fat volume alone.

## Conclusion

Imaging-derived mesorectal fat volume was independently associated with increased operative difficulty and a higher risk of post-operative complications following total mesorectal excision. Compared with BMI and pelvic index, MFV demonstrated superior, although moderate, predictive performance (AUC = 0.655 vs. 0.552 and 0.468, respectively) and may provide additional value for pre-operative risk stratification. Because the multivariable model demonstrated limited explanatory power (Pseudo *R*^2^ = 0.078), MFV should be considered as one component of a comprehensive pre-operative assessment rather than a standalone predictor. Prospective multicenter studies are warranted to externally validate these findings.

## Data Availability

The raw data supporting the conclusions of this article will be made available by the authors, without undue reservation.
