# Supplementary material for: Local fat, local risk: mesorectal fat volume outperforms BMI in predicting rectal cancer surgery outcomes
Source: Front Nutr. 2026 Sep 16;13:1878649. doi: 10.3389/fnut.2026.1878649 (PMC13623603; doi:10.3389/fnut.2026.1878649)
Supplement: Supplementary file 1 [file Table_1.docx]

**Supplementary Table S1. Surgical Characteristics of the Study Cohort (N = 450)**

| **Variable** | **Total Cohort (N = 450)** |
| --- | --- |
| **Surgical approach, n (%)** |  |
| Laparoscopic TME | **320 (71.1%)** |
| Robotic TME | **84 (18.7%)** |
| Open TME | **46 (10.2%)** |
| **Procedure type, n (%)** |  |
| Low anterior resection | **309 (68.7%)** |
| Intersphincteric resection | **45 (10.0%)** |
| Abdominoperineal resection | **96 (21.3%)** |
| **Tumor height category, n (%)** |  |
| Low (<5 cm from anal verge) | **177 (39.3%)** |
| Mid (5–10 cm) | **206 (45.8%)** |
| Upper (>10 cm) | **67 (14.9%)** |
| **Neoadjuvant treatment, n (%)** |  |
| No | **229 (50.9%)** |
| Yes | **221 (49.1%)** |
| **Annual TME caseload** | **Median: 62 cases/year (range, 45–85)** |
